# The Many Facets of Therapy Resistance and Tumor Recurrence in Glioblastoma

**DOI:** 10.3390/cells10030484

**Published:** 2021-02-24

**Authors:** Anshika Goenka, Deanna Tiek, Xiao Song, Tianzhi Huang, Bo Hu, Shi-Yuan Cheng

**Affiliations:** The Robert H. Lurie Comprehensive Cancer Center, The Ken & Ruth Davee Department of Neurology, The Lou and Jean Malnati Brain Tumor Institute, Simpson Querrey Institute for Epigenetics, Northwestern University Feinberg School of Medicine, Chicago, IL 60611, USA; anshika.goenka@northwestern.edu (A.G.); deanna.tiek@northwestern.edu (D.T.); xiao.song@northwestern.edu (X.S.); tianzhi.huang@northwestern.edu (T.H.); bo.hu@northwestern.edu (B.H.)

**Keywords:** glioblastoma, resistance, recurrence, tumor heterogeneity, hypermutation, metabolism, splicing, tumor microenvironment, hypoxia

## Abstract

Glioblastoma (GBM) is the most lethal type of primary brain cancer. Standard care using chemo- and radio-therapy modestly increases the overall survival of patients; however, recurrence is inevitable, due to treatment resistance and lack of response to targeted therapies. GBM therapy resistance has been attributed to several extrinsic and intrinsic factors which affect the dynamics of tumor evolution and physiology thus creating clinical challenges. Tumor-intrinsic factors such as tumor heterogeneity, hypermutation, altered metabolomics and oncologically activated alternative splicing pathways change the tumor landscape to facilitate therapy failure and tumor progression. Moreover, tumor-extrinsic factors such as hypoxia and an immune-suppressive tumor microenvironment (TME) are the chief causes of immunotherapy failure in GBM. Amid the success of immunotherapy in other cancers, GBM has occurred as a model of resistance, thus focusing current efforts on not only alleviating the immunotolerance but also evading the escape mechanisms of tumor cells to therapy, caused by inter- and intra-tumoral heterogeneity. Here we review the various mechanisms of therapy resistance in GBM, caused by the continuously evolving tumor dynamics as well as the complex TME, which cumulatively contribute to GBM malignancy and therapy failure; in an attempt to understand and identify effective therapies for recurrent GBM.

## 1. Introduction

Glioblastoma (GBM) is the most aggressive (WHO grade IV) form of glioma arising from astrocytes or their precursors in the Central Nervous System [1]. It is the most lethal type of glioma with an extremely poor prognosis and a median survival of only 12.1 months [2]. Standard care involves surgical resection followed by radiotherapy along with concomitant chemotherapy with temozolomide (TMZ) and adjuvant doses of TMZ [2,3]. This treatment regimen has increased the median overall survival of GBM patients from 12.1 to 14.6 months in adults [4] and from 7.6 to 9.3 months in elderly patients [5]. However, patients with GBM eventually develop resistance to therapy resulting in recurrent tumors. Thus, an understanding of the diverse mechanisms of resistance is paramount in developing effective treatment regimens against GBM.

Intrinsic resistance to the therapeutic intervention in GBM has long been attributed to the activity of the DNA repair protein O-6-methylguanine-DNA methyltransferase (MGMT). Gliomas with methylated MGMT promoters are more sensitive to TMZ-induced cytotoxicity with a longer median overall survival, as compared to patients whose tumors have unmethylated MGMT promoters [5,6]. However, recent studies show that even MGMT hypermethylated glioma cells show robust expression of MGMT through promoter-independent mechanisms, thereby developing resistance to TMZ treatment [7,8]. Nevertheless, combination therapy using an MGMT inhibitor, O6-benzylguanine (O6BG) alongside TMZ did not show any clinical benefit compared to TMZ alone [9,10]. Moreover, GBM therapy resistance is caused by several other pathways and mechanisms which are either intrinsic to tumor development or are acquired as a result of extrinsic factors such as hypoxia or the immuno-suppressive tumor microenvironment (TME). Factors that are intrinsic to therapy resistance other than MGMT, involve the dynamics of tumor evolution driven by inter- and intra-tumor heterogeneity, development of a high tumor mutation burden resulting in hypermutated tumors, metabolic changes that occur due to manipulation of free radical signaling and oncogenic splicing pathways preferentially expressed in therapy resistant GBM tumors as compared to the normal brain.

Intratumoral heterogeneity is one of the leading causes of therapy failure in GBM and arises due to selective pressures such as clonal competition, nutrient limitation and treatment. Such restricting factors lead to competition between subclones which ultimately results in an ideal microenvironment for tumor growth [11]. Variability in clonal populations is also associated with a diverse mutation load. GBM tumors typically have a low tumor mutation burden (TMB), however a subset of patients develops a high TMB, typically in response to therapy (hypermutated tumors). Previous studies have shown hypermutated tumors to benefit from immune checkpoint therapies, as they offer novel neoantigen signatures [12,13]. However, clinical trials using anti-PD-1 therapy in GBM have shown limited success [14]. Thus, current studies are aimed at understanding the mechanisms by which hypermutation develops and their response to therapy.

The Warburg effect is an intrinsic hallmark of cancer cells, where the tumor cells show a metabolic shift in the generation of ATP from oxidative phosphorylation (OXPHOS) to glycolysis. Cancer cells can utilize aerobic glycolysis, or glycolysis even when oxygen is present, which is less efficient in ATP production as compared to OXPHOS but can utilize glucose in other biosynthetic pathways for the production of lipids, nucleic acids, and some proteins [15]. Similar changes have also been observed in the metabolome of therapy resistant GBM cells wherein different oncogenic variations result in the induction of common metabolic pathways. For example, purine metabolites are known to cause resistance to radiation therapy (RT) by enabling repair of the RT-induced double-strand breaks (DSBs), and thus inhibiting these metabolites alleviates resistance to RT in GBM [16]. Moreover, the oncogenic induction of altered splicing pathways in GBM is another intrinsic regulator of resistance in GBM. Genome-wide transcriptome analysis in GBM has revealed the presence of aberrant AS events in tumors that generate tumor-specific isoforms having enhanced oncogenic activities as compared to normal brain. Such isoforms are also involved in therapy resistance [17,18,19,20]. For example, splicing profiles of glioma subtypes proneural (PN) and mesenchymal (MES) glioma stem cell (GSC) lines have shown significant differences in the genes implicated in the hallmark characteristics of cancer, contributing to PN-MES transition and subsequent tumor heterogeneity and resistance [21].

On the other hand, the hypoxic and immune rich TME extrinsically affects GBM tumorigenesis and response to therapy. Hypoxia, or physiologically low levels of oxygen tension in the tumor, is a major cause of radio-resistance in glioma as RT induces DNA damage but does not maintain its effects [22]. Additionally, in GBM xenografts it has been shown that hypoxic stress increases hypoxia-inducible factor 1 (HIF-1)-mediated induction of the drug efflux transporter ABCB1, leading to chemo-resistance [23]. Furthermore, GBM tumors have a highly immunosuppressive TME, which is the leading cause of immunotherapy resistance in GBM. This is caused by various factors extrinsic to the tumor, such as infiltration of immunosuppressive myeloid cells, expression of checkpoint inhibitors and the dense fibrous tumor stroma that restricts the infiltration of lymphocytes [24]. Other non-neoplastic cells of the TME include the neural precursor cells, tumor vascular niche comprising of endothelial cells, pericytes and vascular smooth muscle cells, astrocytes, and fibroblasts all of which interact with and drive the proliferation of the tumor neoplastic population [25,26]. Thus, cumulative immunotherapy targeting the various barriers of the TME is required to overcome immunotherapy resistance in GBM.

Current studies in the field of recurrent glioma aim at understanding the mechanisms of resistance caused by the aforementioned extrinsic and intrinsic factors. Despite multiple treatment approaches, tumors inevitably relapse after six to nine months of primary treatment and there is no standard treatment for recurrent GBM patients [27]. In a study of 105 patients, who received RT-TMZ treatment, central relapse occurred in 77% patients at the site of the original tumor edge, was the most predominant pattern of relapse [28]. Treatment options include re-resection of tumor, re-irradiation, systemic chemotherapy with the anti-angiogenesis agent bevacizumab, and other experimental approaches through clinical trials. In MGMT-methylated patients relapsing after TMZ treatment, a rechallenge could be proposed [29]. In a study by Mallick et al., 2016, a treatment algorithm has been proposed for recurrent glioma patients based on disease state and patient profile [30]. Though trials have been successful in some patients, no treatment strategy has significantly improved survival of recurrent GBM patients with an average overall survival of less than six months [31,32]. This review aims at understanding the mechanisms of therapy resistance that cause tumor recurrence in glioma, based on recent preclinical and clinical studies.

## 2. Intrinsic Factors of Therapy Resistance

### 2.1. Tumor Heterogeneity-Mediated Therapy Resistance

**Figure 1:**
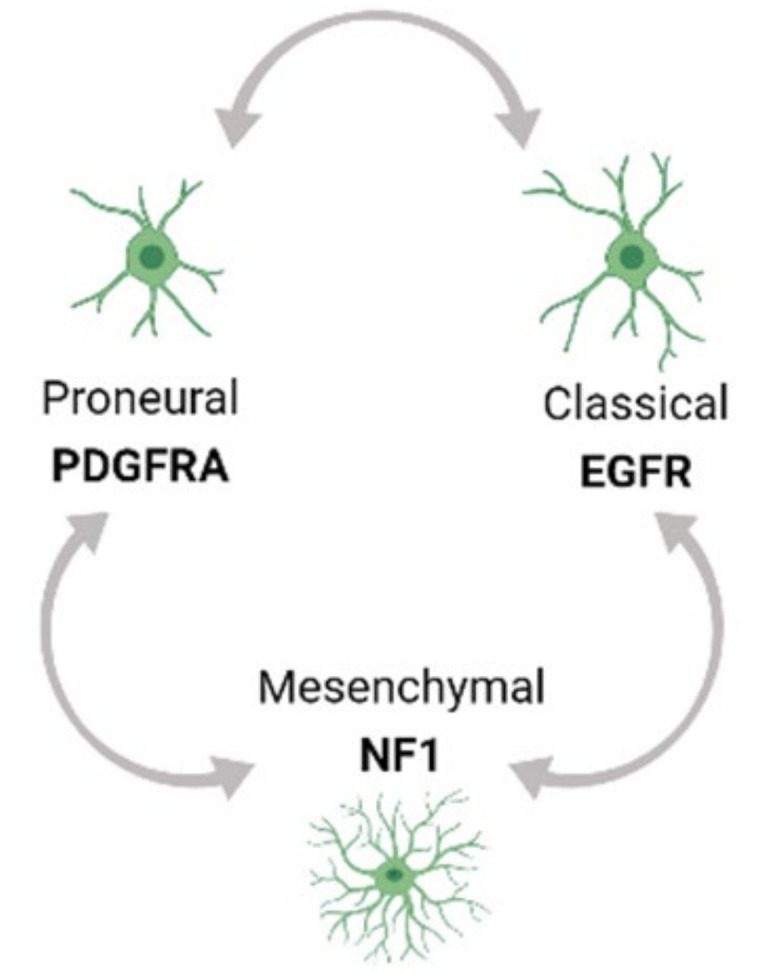
Different transcritptional subtypes of GBM cells and their associated mutations in bold. scRNA seq studies have elucidated three different subtypes of GBM cells namely Proneural (PN), Classical (CL), and Mesenchymal (MES) subtypes manifesting the PDFRA, EGFR and NF1 mutations respectively.

Tumor heterogeneity exists both at the molecular and cellular level and is exhibited within a tumor (Intra-tumor) as well as across different tumors (Inter-tumor). Based on Inter-tumor heterogeneity studies of bulk gene expression analysis from TCGA, glioblastoma (GBM) has been classified into the Proneural (PN), Classical (CL) and Mesenchymal (MES) tumor subtypes [33,34]. Each of these subtypes has a predominant genetic mutation signature. For example, the PN subtype has an enrichment of platelet-derived growth factor receptor A (PDGFRA) mutations while the CL and MES subtypes have a predominance of epidermal growth factor receptor (EGFR) and neurofibromin 1 (NF1) alterations respectively. Moreover, multi-region tumor sampling has shown that different type of cells, and in different ratios, can co-exist within different regions of the same tumor. Thus, regardless of the subtype, each GBM tumor possesses varying populations and types of cells in the TME from all the three subtype tumors, contributing to intra-tumor heterogeneity. Moreover, single cell RNA sequencing (scRNA seq) profiling has shown that the GBM subtypes can inter-change over time and with therapeutic application, thus adding to the complexity of Intra-tumor heterogeneity (Figure 1) [34,35,36].

Further, recent studies using an integrative approach by combining scRNA profiling from a large number of tumor samples with genetic and expression datasets from TCGA have refined these concepts and showed that GBM cells exists in a limited set of developmental states namely (1) neural-progenitor-like (NPC-like), (2) oligodendrocyte-progenitor-like (OPC-like), (3) astrocyte-like (AC-like), and (4) mesenchymal-like (MES-like) states. These states are driven by genetic alterations in cyclin dependent kinase 4 (CDK4), PDGFRA, EGFR, and NF1 genes respectively, that favor a particular developmental state, confirming the modulation of GBM cells by GBM genetic drivers. Further, lineage tracing of uniquely barcoded single cells in vivo has shown that these cells exhibit plasticity, and a single cell type has the potential to convert into all other cell types influenced by the in vivo TME, collectively contributing to the heterogeneity in GBM and rendering GBM therapeutically resistant [36,37].

Such divergent evolution of sub-clonal populations with distinct mutation signatures and cell types in different ratios in the same tumor (intratumoral heterogeneity) is the root cause of failure for multimodal therapies including RT, chemotherapy, and other targeted therapies. Though, therapeutic treatments can target and destroy the treatment-sensitive cell clones or fractions of subpopulations, outgrowth of treatment-resistant clones or populations (both intrinsic and acquired resistance) are responsible for therapy-resistance and ultimately tumor recurrence [38]. Studies profiling low- & high-grade gliomas (LGG & HGG) and their paired recurrent tumors have shown temozolomide (TMZ)-therapy to drive such treatment-resistant clones as the recurrent counterparts were found to bear a specific TMZ-induced mutagenesis signature [39,40,41]. Both linear and divergent models of clonal evolution have been attributed to tumor recurrence and subsequent resistance. While some GBM recurrences bear driver mutation signatures such as p53 already present in the primary/initial tumor (linear evolution), other recurrent tumors show a more branched divergent evolution wherein the acquired mutations driving the tumor recurrence are not present in the primary tumor [39,41,42]. To summarize, when a normal cell acquires sequential genetic mutations and evolves into a tumor initiating cell, it expands and further evolves into different sub-clonal populations giving rise to intratumoral heterogeneity. External stresses such as application of therapies could lead to selection of early or late clonal populations in these evolution processes or generate therapy-selected subclones which could be resistant to current or different therapies such as RT. The therapy-resistant clones then seed the recurrent tumor which is comprised of a new heterogenous TME having a distinct clonal population from the original tumor and harbors a distinct set of genetic mutations which are both new and common to the initial tumor [38].

Meta-analysis of clinical trials in GBM has documented the failure of monotherapies in GBM owing to the complex intratumoral heterogeneity and has highlighted the need to develop newer multimodal therapies to treat this complex disease. Unfortunately, recently tested monotherapies have been met with limited success in clinical trials. For example, monoclonal antibodies targeting programmed cell death protein 1 (PD-1) protein and its ligand programmed death-ligand (PDL-1) to enhance the cytotoxic activity of CD8^+^ T cells has been found to be successful in other cancers such as melanoma and non-small cell lung cancer but failed to show success in GBM as reported by the CheckMate phase III clinical trial (NCT02017717) [43]. Moreover, even combination therapies coupling anti-PD-1/PDL-1 with other targets which have proven to be successful pre-clinically have not progressed ahead to clinical trials [44,45]. The clinical trial targeting EGFRvIII, a constitutively active mutant of EGFR, through the vaccine rindopepimut (phase III ACT IV clinical trial) did not show any improvement in patient survival in GBM, possibly due to the loss of EGFRvIII expression upon therapy or in recurrent GBM [46]. Such studies thus emphasize a deep understanding of intratumoral heterogeneity in GBM and stress the need of further understanding of the underlying tumor biology that will lead to effective multimodal therapies. Furthermore, a novel small molecule inhibitor, BGB324, has been tested pre-clinically in immunocompromised mice with GSC-derived MES-like GBM tumors to target the TAM (Tyro-3, Axl, and Mer) receptor tyrosine kinase family member Axl, thereby disrupting it’s signaling with ligand protein S (PROS1), has significantly improved survival and is thus under investigation in recurrent GBM models. If successful, this approach might be promoted to clinical trials [47]. Thus, coupling this molecule with RT, which has the strongest impact on proneural cells, and TMZ which showed efficacy in rapidly proliferating CL GBM cells, might be addressing the complex issue of tumor heterogeneity in GBM [48].

### 2.2. Role of GBM Tumor Models in Studying Therapy Resistance

In addition to the complex heterogeneity of GBM tumors at the genetic and developmental level, there exists a unique immune TME. In order to have a deep understanding of the tumor biology, GBM models are needed which faithfully recapitulate potential GBM states and their association with the TME. Serum-free glioma spheres is a widely used in vitro GBM model which have been categorized to be both radio- and chemo-resistant, but it is not clear if a radio-chemotherapy would lead to selection of subclones which would lead to recurrence [49,50,51]. Also, the lack of tumor architecture and TME interactions in these in vitro models limits its usefulness. Genetically engineered mouse models (GEMMs) are derived from combined mutations in the primary GBM tumor that drive tumor growth. The GEMMs recapitulate the tumor architecture and are useful in studying signaling mechanisms linked to driver mutations. However, in a GBM tumor there are multiple mutations linked to tumorigenesis and hence their interdependence cannot be examined in the GEMMs [38]. Patient-derived xenograft (PDX) models on the other hand provide a complete mutational profile that can be stably passed in vivo, possess a TME that can form heterogenous cells and are thought to recapitulate the histopathological features of the parent GBM [52]. However, recent studies have shown PDX tumors to follow the trajectory of a mouse-specific tumor evolution, thus jeopardizing their usefulness for therapeutic modeling [53].

These challenges have been met with the recently developed tumor organoid (TO) [54] model and the cerebral organoid glioma (GLICO) model wherein patient-derived GSCs home towards human embryonic stem cell (hESC)-derived cerebral organoids and deeply invade and proliferate within the host tissue to form tumors that phenocopy human tumors in clinic [55]. Moreover, cancer avatar tumor organoid models derived from genetically engineered human pluripotent stem cells (hiPSCs) are shown to form secondary tumors that resemble patient tumor characteristics including tumor heterogeneity. Introduction of GBM-associated genetic driver mutations in hiPSCs (GBM avatars) has been shown to result in the formation of high-grade gliomas that recapitulate the authentic cancer pathobiology [56]. Further, recent studies including three-dimensional culture conditions and scRNA seq has enabled the generation of GBM organoids (GBOs) in which unlike the above organoid models, pieces of GBM around 1 mm in diameter are cultured instead of dissociating them into single cells, this retains the cell-cell interactions without the presence of an extracellular matrix. scRNA seq, histopathological analysis, and molecular profiling have shown that GBOs faithfully recapitulate the cellular and molecular profiling of GBM patient tumors and represent both inter- and intra-tumor heterogeneity. These organoid tumor models also recapitulate other features of GBM tumors which are difficult to model in in vitro conditions such as hypoxic gradients and tumor heterogeneity [57,58].

Current in vitro and in vivo GBM models are derived from primary tumor tissues obtained at initial diagnosis to study pathway signaling in tumorigenesis and are extrapolated for their role in recurrent tumors. However, the divergent evolutionary nature of the tumor shows that recurrent tumors are molecularly different from the primary/initial tumors. Also, most animal models do not reveal the progression of tumor from a treatment naïve-initial tumor state to a treatment refractory-relapse tumor condition. Hence models which recapitulate the recurrent TME are required.

### 2.3. Hypermutation-Induced Therapy Resistance

Owing to the subsequent resistance to therapy and inevitable tumor recurrence, scientists resorted to large scale longitudinal analysis to compare pre- and post-treatment tumor samples using high-throughput exome and transcriptome sequencing [11,39,40,41]. Such analysis revealed recurrent tumor types of two genomic outcomes–*hypermutant* and *non-hypermutant* [59]. Hypermutated tumors were categorized by an increased load of tumor burden, having >500 mutated genes per tumor, whereas the non-hypermutated recurrent tumors had only ~50 mutations on average [39,41]. Moreover, hypermutated recurrence arises only in TMZ-treated patients and were enriched for a unique TMZ-specific mutation signature of C>T (G>A) transitions. Also, most of the hypermutated tumors gain mutations in genes encoding DNA mismatch repair (MMR) proteins, and primarily MutS homolog 2 (MSH2) and MutS homolog 6 (MSH6) [41]. The association of MMR mutations and hypermutation in glioma has been studied for a long time [60,61], however, they have not been functionally categorized and thus their role in causing hypermutation is not clear. A recent study profiling 93 paired (primary and recurrent) glioma samples has shown that all eight of the recurrent tumors which had MMR inactivation resulted in hypermutation [41]. Moreover, in a much larger study involving 10,294 glioma patients, identification of hypermutation driver mutations has shown that MMR mutations are the most enriched ones (~91.2%) among a total of 36 enriched genes [40].

Next, earlier studies have shown that TMZ is a direct cause of hypermutation in recurrent gliomas as seen by the accumulation of a TMZ-specific mutational signature in these tumors [39,62]. However, whole-exome and transcriptome studies in the paired tumor samples have shown that only 17% of patients (17 of 100) in one study and 25.8% patients (58 of 225) in another study, who had been subjected to TMZ treatment, resulted in hypermutant recurrent tumors [40,41]. Thus, TMZ treatment in glioma might not always result in hypermutation [40]. Interestingly, in the aforementioned studies, 16 of 17 patients [41] and 91.2% of the 58 hypermutated patients [40] had MMR gene mutations, likely suggesting that TMZ-treated gliomas that gain MMR inactivation mutations, universally evolve to hypermutated recurrent tumors, while those lacking these potential driver mutations resulted in non-hypermutant recurrent tumors.

Further, the study by Touat et al., 2020 which studied the mutational burden by profiling 10,294 gliomas, found that hypermutant tumors or tumors with high mutation burden have a worse overall survival as compared to non-hypermutant tumors in different types of glioma including 1p/19q co-deleted oligodendrogliomas, isocitrate dehydrogenase 1/2 (IDH1/2)-mutant astrocytomas and IDH1/2 wild-type GBM [40]. However, contrary studies from the Glioma Longitudinal Analysis (GLASS) Consortium analyzing a smaller yet sizable cohort of 222 glioma patients, reported no difference in the overall survival of patients with hypermutant vs non-hypermutant recurrences. The driver mutations manifested in the original tumor were retained at recurrence with a slim possibility of developing or acquiring new mutations at recurrence and the rate of hypermutation was different across different glioma subtypes [11]. Both studies did longitudinal analysis of paired original and recurrent tumors samples using DNA-sequencing datasets, however, report opposing findings.

Studies in cancers with high tumor mutation burden are now focusing on exploiting the increased neoantigen burden in such cancers to enhance immunogenicity. Cancers with MMR deficiencies have been approved to be treated clinically by the PD-1 blocker pembrolizumab [63,64]. However, MMR deficiency generally occurs at the initiation of the tumor, for example in colorectal cancer (CRC), unlike in glioma, where MMR deficiency occurs later due to post-treatment recurrence, and hence the immune regulation and response to immunotherapy in glioma may differ from other cancers with MMR deficiencies. In an attempt to find an association between MMR deficiency and immune infiltration, Touat et al., 2020 found that while there was a high T-cell infiltration in CRC patients with MMR deficiency, as compared to their MMR-proficient counterparts, both MMR-proficient and post-treatment MMR–deficient glioma patients had reduced T-cell infiltration. Further, PD-1 blockade did not show any increase in survival or any histopathological changes in glioma patients, thus suggesting a difference in the mutational landscape of hypermutated gliomas as compared to other types of immune-responsive hypermutated cancers [40]. However, another study has shown clinical benefit in a pair of siblings with recurrent GBM having biallelic mismatch repair deficiency, wherein treatment with the PD-1 inhibitor nivolumab showed significant clinical and radiological responses [12].

Thus, other strategies of chemotherapy are warranted which retain sensitivity in recurrent tumors and do not lead to such a high tumor mutation burden which ultimately leads to therapy-resistance and reduced overall survival as compared to tumors with low mutation burdens. Mathematical modeling studies using data from TMZ-treated patients or with a nitrosourea drug called CCNU, predicted acquired resistance in 51% of LGG patients treated with TMZ. However, none of the CCNU-treated patients were classified into the acquired resistance category, suggesting that TMZ paradoxically increases tumor progression in a subset of patients and hence identification of such a risk group is of high importance [65]. This finding has been corroborated by the Touat et al., 2020 study where the authors show that all MMR-deficient models that were resistant to TMZ, were sensitive to CCNU, suggesting that hypermutant gliomas might still be sensitive to DNA damaging agents other than TMZ [40].

### 2.4. Splicing-Mediated Therapy Resistance

**Figure 2:**
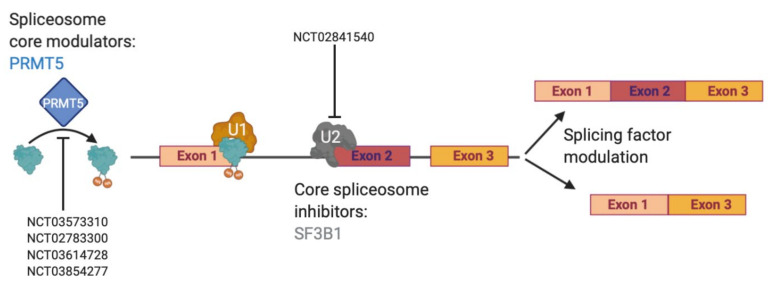
Splicing can be modulated at multiple levels. Pre-splicing, modifying enzymes, like the methyltransferase PRMT5, can be inhibited preventing spliceosome assembly. Core spliceosome components, like SF3B1, can also be inhibited leading to unproductive splicing. Isoform modulation can also be a target of alternative splicing to switch to a less oncogenic protein isoform. Clinicaltrials.gov identifiers (NCT) are included where small molecule inhibitors or modulators are being tested in humans.

Currently, effective therapeutic targets for GBM are severely lacking as the clinical standard of care has not changed since the Stupp protocol was put into place in 2005 [4]. However, dysregulated RNA alternative splicing (AS) has gained attention in the past 15 years as sequencing has become more affordable and commonplace. AS is the process of removing introns and joining together exons [66] in ways that are dictated by the spliceosome and many accompanying splicing factors [67,68]. The spliceosome is a large protein complex made up of multiple small nuclear RNA (snRNAs)–U1, U2, U4, U5 and U6–that combine with over 80 proteins to create RNA-protein complexes known as small nuclear ribonucleoproteins (snRNPs, pronounced “snurps”) [69,70]. Although few mutations in splicing factors or RNA binding proteins (RBPs) are found in gliomas, dysregulation of AS in glioma has been described [71]. A large number of AS isoforms with oncogenic functions in glioma has also been reported [8]. Furthermore, the U2 snRNP has received a lot of attention due to a number of mutations in its protein member splicing factor 3B subunit 1 (SF3B1) in chronic lymphocytic leukemia and uveal melanoma [72,73,74,75]. Many drugs have now been designed to specifically target SF3B1, including in glioma [76,77]. The SF3B1-targeting compounds spliceostatin A and sudemycin C1 have been shown to specifically target glioma stem cells, which are notoriously known for being therapy resistant, over normal neural stem cells [78]. A third SF3B1-targeting agent, palidomide B, has also been shown to be effective against multiple central nervous system (CNS) cancer lines, even before its target was fully realized [79]. However, targeting the spliceosome may be tricky as there must be a large enough therapeutic window to show a cancer-specific effect and an ability to cross the blood brain barrier. Nevertheless, multiple clinical trials have been designed to answer this question [80]. As we await the results of direct spliceosome inhibition, other indirect ways to target splicing remain through splicing factors and isoform modulation (Figure 2).

Accumulated studies have shown that splicing factors are dynamically expressed between normal brain and gliomas [81]. One of the larger studies evaluated over 1,500 RBPs, finding 223 and 225 overexpressed between GBM and normal brain and glioma stem cells (GSCs) and neural stem cells (NSCs), respectively, with 58 overlapping between GBM and GSCs [82]. As such, targeting overexpressed RBPs has become an active area of research in GBM. Overexpression of SRSF3 in GBM patient samples and GSCs was shown to effect exon usage of multiple mitosis-related genes, where knockout normalized exon usage and increased overall survival in mouse models [17]. In a high throughput screen of TMZ-treated GL261 cells, Braun et al discovered a dependency on the arginine methyltransferase PRMT5, showing its role in regulating detained introns. They went on to show that PRMT5 knockdown or inhibition could reverse this oncogenic splicing pattern and further created a gene-based score for PRMT5 inhibition sensitivity [71]. Following this trend, post TMZ treatment, RBM11 was shown to increase in expression where it was then packaged into apoptotic vesicles that were taken up by surrounding surviving cells, which changed overall splicing patterns and decreased TMZ drug sensitivity [83]. These studies show that targeting splicing factors and RBPs in glioma is still an active and attractive area of research for therapeutic development.

Another indirect way to target splicing is through modulation of specific protein isoforms. Zhou et al focused on the downstream targets of SRSF1, which is upregulated and a poor prognosis predictor in GBM and found that SRSF1 upregulation led to an increase of the myosin IB full length isoform (MYO1B-fl). This isoform was membrane-bound and promoted GBM cell proliferation, invasion, and survival [84]. Similarly, an increase in SRSF6 phosphorylation led to an increase in the estrogen-related receptor beta 2 (ERRb2), which was also membrane-bound and inhibited GBM cell migration, invasion, and antagonized the nuclear-localized short form (ERRbsf) isoform, dampening its transcription regulatory functions. Using both an inhibitor for SRSF6 phosphorylation, TG-003, and an ERRb agonist, DY131, the isoform ratio was shifted to favor the ERRb2 isoform, which could be activated by DY131 to promote cell death [85]. EGFRvIII effects isoform regulation through upregulating heterogeneous nuclear ribonucleoprotein A1 (hnRNPA1). hnRNPA1 promotes the splicing of Delta Max, a Myc interacting protein, which augments EGFRvIII expression and promotes GBM cell proliferation in vitro [86]. Overall, a better understanding of dysregulated AS in GBM is warranted and may lead to better therapeutic options in the future.

### 2.5. Metabolism as a barrier to RT and TMZ treatment

Radiation therapy (RT) and temozolomide (TMZ) treatment have been the two main workhorses in treating GBM [87]. However, while they work upfront, resistance is rapid and being able to better understand this resistance is necessary to develop second-line therapeutics [88]. The conventional mechanism of action for radiation is dependent on reactive oxygen species (ROS) that cause DNA damage resulting in oxidative stress [89]. Normal cell metabolism favors OXPHOS where the final glycolysis product, pyruvate, is shuttled into the mitochondria where electrons are transferred through the electron transfer chain (ETC) to maximize production of ATP. However, this process is dependent on oxygen and in low oxygen, or hypoxic conditions cells will turn to lactic acid fermentation, or anaerobic glycolysis thus increasing the production of ROS [90]. Therefore, an attractive avenue to increase RT sensitivity, or re-sensitize to RT, would be to increase intracellular ROS or deplete antioxidants. Post RT treatment, antioxidants levels, like ascorbic acid and glutathione (GSH), are decreased, with an increase of ATP and GTP [91]. With this knowledge, experiments have been focused on manipulating the levels of glutathione and ascorbic acid to treat radioresistant tumors [92]. One method that is currently in trials is high dose ascorbic acid, which has been shown to markedly increase intracellular ROS and is thought to inhibit glycolysis, disrupt labile iron metabolism, and induce double strand breaks (DSBs) [93,94,95]. In addition, high ascorbic acid treatment showed radio-sensitization of GBM to be a ROS-dependent cell death [96]. The combination treatment of radiation and ascorbic acid is currently being tested in clinical trials (NCT02344355).

ROS modulation in IDH mutant (IDHmut) tumors can also re-sensitize to RT through targeting glutamine metabolism. Glutaminase (GLS) catalyzes the hydrolysis of glutamine to glutamate (and ammonia), which is one of the three amino acids necessary to make the antioxidant GSH [97]. As (R)2-hydroxyglutarate (HG), the metabolite made by IDHmut, inhibits the 2-oxoglutarate-dependent transaminases branched chain amino acid transaminase (BCAT)-1 and BCAT-2, these cells become dependent on GLS for glutamate and subsequent GSH production [98]. In this way, GLS inhibition by CB-839 has been shown to re-sensitize IDHmut gliomas to RT both in vivo and in vitro by depleting the antioxidant GSH [98]. Currently, the combination of CB-839, radiation, and TMZ is recruiting IDHmut patients for a clinical trial (NCT03528642).

Increasing mitochondrial ROS (mROS) has also been investigated to sensitize cells to RT. Post-radiation, the Hypoxia Inducible Factor 1α (HIF1α) target gene pyruvate dehydrogenase kinase (PDK) has been shown to be upregulated. PDK is a regulator of pyruvate fate, where inhibiting PDK leads to a decrease of lactate production and increases both glucose oxidation and mROS [99]. This effect has been shown both in vitro and in vivo with concurrent radiation and in hypoxic conditions [100]. Furthermore, it has been shown that a PDK inhibitor, dichloroacetate (DCA), can cross the blood brain barrier (BBB) and was well tolerated in patients [101]. However, more research needs to be completed to determine the efficacy of DCA in prolonging patient survival. While targeting tumor metabolic changes induced by RT may be a potential therapeutic avenue in the future, recent work has also shown that mice that were pre-irradiated before tumor cell engraftment showed a decreased response to post-engraftment radiation treatment [91]. This study shows the importance of understanding the effects of radiation as most recurrent gliomas will recur in a pre-irradiated microenvironment. Therefore, to design better second line treatments, the effect of previous radiation needs to be considered.

However, RT is combined with the chemotherapeutic agent TMZ as the current standard of care treatment. TMZ is an alkylating agent that induces DNA damage, which can be reversed by the DNA repair protein methyl guanine methyl transferase (MGMT) [102]. Tumors expressing MGMT are intrinsically resistant to TMZ, however others become resistant post TMZ treatment as chromatin changes can allow MGMT to become expressed [103]. As this pathway has been exhaustively targeted, another potential target are the abnormal metabolic changes due to TMZ treatment.

While most studies focus on nuclear DNA damage caused by TMZ, Oliva et al investigated the effect of TMZ on mitochondrial DNA (mtDNA). They found a decrease in total mtDNA, as well as large deletions that effected mtDNA integrity post TMZ treatment. As mtDNA codes for electron transport chain (ETC) proteins, they went on to show acquired TMZ resistance in both GBM cell lines and clinical samples showed a remodeling of the ETC with a decrease in Complex I and V, and an increase in Complex II-III, and IV in mitochondria [104]. This change in ETC components was corroborated in a second TMZ-acquired resistant model where Complex I, II, and IV were upregulated [105]. Furthermore, in paraganglioma, an inactivating mutation in Complex II has been associated with increased TMZ responsiveness, underscoring the importance of the ETC in TMZ efficacy [106,107]. To this end, multiple studies have looked to repurpose the type II diabetes drug, metformin, to target TMZ-resistant cells [108]. While the actual mechanism of action is uncertain, metformin treatment was shown to modulate anabolic mitochondrial metabolism and induce cell cycle arrest and cell death, re-sensitizing TMZ-resistant cells to TMZ treatment [109]. Another potential metabolic target is fatty acid oxidation, as Caragher et al recently showed that TMZ induces a dependence on endogenous fatty acid oxidation, that was enriched in CD133+ GSCs and the proneural GBM subtype [110].

TMZ has also been shown to increase the expression of aldehyde dehydrogenase 1 family member A1 (ALDH1A1) via the long non-coding RNA TP73-AS1. ALDH1A1 has been used as a marker for TMZ-resistant GSCs, where inhibition of ALDH1A1 increased responsiveness to TMZ treatment [111]. Clinical trials are currently investigating the therapeutic potential of ALDH1A1 inhibitors in combination with TMZ and RT [112]. As the two main treatments for GBM-TMZ and RT-both affect tumor metabolism, further research should be completed to discover metabolic dependences with modulators that can cross the BBB and give better therapeutic options for GBM patients in the future.

## 3. Extrinsic Factors of Therapy Resistance

### 3.1. Hypoxia-Induced Therapy Resistance

Hypoxia, or lack of oxygen, is a common feature of fast-growing tumors like GBM as they quickly outgrow their vasculature and subsequent nutrient supply [113]. Normal brain oxygenation was shown to be ~40 mmHg by Eppendorf needle electrode, whereas GBM clocked in at ~10 mmHg where 0-10 mmHg was shown to correlate with RT resistance [114,115]. Hypoxic tumors also show a greater resistance to chemotherapy, and because of their lack of properly formed vasculature, drug profusion is difficult leading to a dismal patient prognosis [116]. TMZ treatment has been shown to increase the expression of HIF1α, a transcription factor that regulates genes involved in dedifferentiation, genomic stability, metastasis, and maintenance of stem cells, among others [117]. CD133, a surface marker of glioma stemness, is also increased in hypoxic gliomas where its upregulation is proposed to be through HIF signaling [55]. Hypoxic conditions have also been shown to decrease the response of GBM cells to the standard of care treatment for GBM, TMZ [118].

HIF1α is able to work in conjunction with the oncogene c-Myc to alter tumor metabolism [119]. c-Myc interacting with HIF1α increases the expression of the glucose transporter GLUT1, which allows for an influx of glucose, and hexokinase 2 (HK2), the enzyme necessary for the first step of glycolysis, thereby directing the cells towards glycolysis [120]. This switch to glycolysis has been shown to help cancer cells adapt to hypoxic conditions by increasing the synthesis of ATP and biomolecules [121]. HIF1α is also controlled by the PI3K pathway, where loss of phosphatase and tensin homolog (PTEN), a common feature of GBM, can increase HIF1α activation and where PI3K inhibition decreases HIF1α expression [122].

Reoxygenation via hyperbaric chambers [123] or misonidazole and nimorazole–oxygen mimetics–have been moderately successful in increasing radiation sensitivity [124]. However, hypoxia-activated compounds like quinones, N-oxides, and tirapazamine, while able to decrease tumor hypoxia, were poorly perfused and had toxic side effects which greatly limited their clinical use [123]. Therefore, the current strategy to increase oxygen consumption within the tumor is by targeting the mitochondrial oxygen consumption rate (OCR). Inhibition of the electron transport chain (ETC) at the mitochondrial membrane, which requires oxygen in the final step, allows for an increase in tumor oxygen concentrations and re-sensitization to radiation [125]. Inhibitors to complex I (biguanides), complex II (alphatocopheryl, lonidamine, and VLX600), complex III (atovaquone), and complex IV (VLX600 and arsenic trioxide) have been tested [126]. While the complex I biguanide, metformin, reduced OCR by ~10-20%, the complex III inhibitor atovaquone showed a decrease of ~80% in OCR [127]. Though not yet tested in GBM, atovaquone has shown radiation sensitizing effects in hypopharyngeal carcinoma [128] and is currently being tested as a hypoxia modifying agent in a non-small cell carcinoma clinical trial in combination with radiation (NCT02628080). Importantly, atovaquone is able to cross the BBB, warranting more research into this potentially promising therapeutic avenue for drug resistant gliomas [129].

### 3.2. Mechanisms of Immunotherapy Resistance

The GBM TME is a diverse milieu of both neoplastic and non-neoplastic cells in which the tumor cells grow and develop diversity within the tumor. GBM tumors differ from other solid tumors as they are located in the brain, an immune-privileged organ, where the infiltration of the peripheral immune cells is restricted due to the brain-blood-barrier (BBB). However, the BBB is disrupted by inflammation, rapid expansion of GBM tumors, and tumor infiltration by immunosuppressive immune cells from blood circulation categorizing it as a ‘cold’ tumor [130]. Flow cell analysis of clinical GBM biopsy samples have shown that T cells account for only 0.25% of the GBM tumor of which CD8^+^ cytotoxic T cells, which are the effector killer T cells, account for only one-fourth of the total CD3^+^T cell population. Also, these T cells are less responsive to anti-CD3^+^ stimulation in vitro as compared to their healthy counterparts, indicating an immunosuppressed state within the GBM TME [131]. Moreover, a study that characterized the TME across 33 cancer types identified six different immune subtypes with unique signatures and revealed that LGG and GBM were the most prevalent in the ‘immunologically quiet’ immune subtype which exhibited the lowest number of lymphocytes and the highest number of macrophages dominated by the ‘Tumor Associated Macrophage’ population [132]. Additionally, other studies have shown that immunosuppressive cytokines and chemokines such as transforming growth factor beta (TGF-β), interleukin 10 (IL-10), prostaglandin E2, and immune cells like immunosuppressive natural killer T (NKT) cells, T/B regulatory cells (T/Breg), tumor-associated macrophages/microglia (TAMs), and myeloid-derived suppressor cells (MDSCs) create an immunosuppressive microenvironment in glioma, supporting pro-tumorigenic activities which lead to tumor progression [133,134,135].

The above mechanisms pose an intrinsic resistance in GBM. Thus, appropriate target antigens must be identified and selectively delivered to overcome immunotherapy resistance. Upregulation of immune checkpoint molecules are commonly observed in solid tumors; however, their expression increases dramatically under the pro-tumorigenic immune-TME, wherein the immunological milieu co-opts the tumor cells to drive tumor progression [24]. Immune checkpoint inhibitors which have shown clinical success are targeted against the immune checkpoint molecules PD-1 and cytotoxic T-lymphocyte-associated protein 4 (CTLA-4). These checkpoint inhibitors are expressed by T cells and perform inhibitory functions by interacting with their corresponding ligands that are expressed on both the tumor and TAMs, to release cytokines, leading to inhibition of T cell function, polarization of macrophages to a pro-tumorigenic state and thus tumor progression [136]. Antibodies against the PD-1 and CTLA-4 checkpoints have been successful in several solid cancers. However, in a phase III clinical trial of Nivolumab (monoclonal antibody against PD-1) in GBM, only 8% of patients responded in the trial (clinical trial NCT02017717). To this end, alternative checkpoint receptors such as T-cell immunoglobulin and mucin domain-3 (TIM-3) have been found to be upregulated in tumors with delayed resistance to PD-1 blockade and combinatorial therapy against PD-1 and TIM-3 has increased efficacy in pre-clinical models [137]. Thus, current clinical efforts are aimed at targeting additional checkpoints to overcome the resistance to PD-1 or CTLA-4 blocking antibodies [24].

TAMs are the dominant infiltrating immune population in glioma constituting around 30–40% of the total tumor volume [25,132,138]. Studies using scRNA seq analyses have revealed that the MES subtype of GBM has the maximum infiltration of the myeloid cell population [138,139] which has been associated with a poor prognosis in GBM [140]. In GEMMs of platelet derived growth factor subunit B (PDGFB)-driven glioma, it has been shown that TAMs derived from bone marrow-derived monocytes constitute the major proportion (up to 85%) of TAMs in the GBM TME, whereas those derived from brain-resident microglia constitute only 15% of the total TAM population in GBM [141]. Furthermore, a scRNA seq study showed that blood-derived TAMs up-regulate immunosuppressive cytokines, exhibit altered metabolism as compared to microglia-derived TAMs, and that the gene-signature of blood-derived TAMs correlates with an inferior survival in patients with glioma [142]. The GBM TME attracts TAMs to the tumor and polarizes them to an anti-inflammatory or pro-tumorigenic ‘M2′-like state, thus current studies are aimed at inhibiting their recruitment or survival in the TME, allowing for their functional re-education to an anti-tumor ‘M1′-like state, or targeting the tumor using monoclonal antibodies that elicit macrophage-mediated phagocytosis and intracellular destruction of cancer cells [143].

Additionally, a recent study showed that kynurenine, a metabolite produced by glioma cells, attracts TAMs to the TME by up regulating the expression of a transcription factor aryl hydrocarbon receptor (AHR) in TAMs thus regulating their function that in turn reduces T cell immunity by producing the metabolite adenosine in conjunction with CD73. The expression of AHR is the highest in GBM-relative to lower grade gliomas and is independently correlated with poor patient prognosis, thus making AHR an attractive target for GBM immunotherapy [144]. The cytokine CSF-1 (colony-stimulating factor-1) has been shown to be critical in the function and survival of TAMs. Inhibition of the CSF-1 receptor (CSF-1R) to target TAMs has been shown to regress tumor formation and increase survival in GBM mouse models. Moreover, although inhibition of CSF-1R through blocking antibodies showed a high response rate in a preclinical GBM models, tumors recurred in >50% of the mice due to acquired resistance through re-activated phosphoinositide 3-kinase (PI3K) signaling [24]. Additionally, a phase II clinical trial with the CSF-1R oral inhibitor PLX3397 failed to improve survival in 37 recurrent GBM patients [145]. Lastly, the cytokine interleukin 2 (IL-2) has been found to convert TAMs from a pro-tumorigenic mode to a tumor inhibiting state and attempts are underway to deliver them to the TME using nanoparticles [146]. Moreover, studies using CD47-blocking antibodies have shown promise in preclinical GBM models [24] but their clinical relevance for treating GBM is yet to be determined.

Genetic alterations induced by immunological pressures are known to develop into acquired resistance in the tumor that diminishes the effect of immunotherapy and ultimately results in delayed treatment failure. In a recent clinical study, 66 patients with recurrent GBM who were treated with PD-1 blocking antibodies were longitudinally profiled before and after treatment. Seventeen patients were categorized as responders based on tumor regression and inflammation. Genomic and transcriptomic studies revealed that the responders were enriched for gene mutations in the mitogen-activated protein kinase (MAPK) pathway and showed a branched pattern of evolution resulting from the elimination of neo-epitopes whereas non-responders were enriched for PTEN mutations associated with immunosuppressive gene signatures and non-clonal evolutionary patterns. Thus, the clinical response to anti-PD-1 treatment in GBM is associated with specific molecular patterns and clonal evolution during treatment, lack of which develops an acquired resistant state in the glioma tumor, thus resulting in therapy failure [24,147].

Finally, the role of neurons and neuronal precursors in driving glioma progression has been of recent interest in the field. In an interesting study done with glioma-bearing mice, where mice were housed either under standard conditions or in enriched conditions mice in larger numbers, in cages with toys, it was found that the environmental cues induced the activity of phagocytic macrophages in the TME by brain-derived neurotrophic factor (BDNF) and pro-inflammatory cytokines by NK cells [148]. Another study has shown the presence of neuron-glioma interactions through the formation of electrochemical synapses involving the α-Amino-3-hydroxy-5-methyl-4-isoxazolepropionic acid (AMPA) receptor, and that pharmacological or genetic blocking of this electrochemical stimulation inhibits the growth of mouse xenografts and improves survival [149]. Further it is shown that such neural-glial signaling is involved in breast-to-brain metastasis through N-methyl-D-aspartate (NMDA) receptor mediated neuronal signaling [150]. Interestingly, different neuronal subtypes display variable activities. The cortical pyramidal neurons drive tumor progression while the Gamma-aminobutyric acid (GABA)-ergic interneurons reduce glioma cell proliferation [151]. Such synaptic driven brain tumor progression could further be explored in resistant glioma patients to study their role in tumor recurrence.

## 4. Conclusions

**Figure 3:**
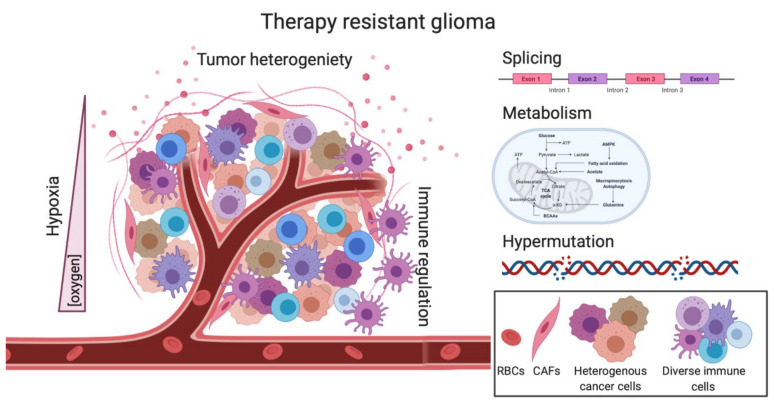
Complex pathways underlie the therapeutic resistance of GBM. The decrease of oxygen within in the tumor can facilitate hypoxia-induced signaling which can render cells less sensitive to treatment. Tumor heterogeneity can complicate therapeutic response as not all clones are targeted equally by standard treatments. Immune regulation is severely dampened in GBM, creating a “cold” TME. Splicing changes via RBPs or splicing factors can affect isoform outcome leading to dysregulated AS. Metabolic changes induced by TMZ or RT are able to change metabolism in both the tumor cells as well as the TME, creating a therapeutic-resistant microenvironment. TMZ-induced hypermutation can create a hypermutator state in which patient outcome is correlated with a worse overall survival, as compared to other cancers. Overall, many pathways play a role in GBM therapeutic resistance, and all should be investigated for better second-line treatments. RBPs, RNA binding proteins; AS, Alternative splicing; RBCs, red blood cells, CAFs, cancer-associated fibroblasts.

Despite the ongoing work and advancements made in better understanding some of the molecular mechanisms underlying GBM disease and progression, more work is needed to continue to make progress in understanding this universally fatal disease. Unlike other cancers, GBM does not have standard and effective second-line therapeutic options. Here, we outlined areas such as heterogeneity, hypermutation, metabolism, splicing, hypoxia and the immune system, that are being actively investigated to hopefully provide successful second-line therapies (Figure 3). 

While TMZ is currently the only effective 1st line therapeutic option, along with surgery and radiation, we show how in a section of GBM patients, TMZ treatment can create a hypermutator state which has been correlated to worse overall survival in patients. While in other cancers, this hypermutator state has been shown to induce a “hot” immunologic tumor, this effect is not noted in GBM, and the benefits of immunotherapies have been underwhelming so far. However, new immune cell receptor targets are being developed. TMZ also affects the metabolic state of both the tumor and the TME that can create a radio- and chemo-resistant environment through both hypoxia and other metabolic intermediates. Direct and indirect targeting of splicing factors may be a promising future direction where the outcome of many clinical trials is eagerly being awaited. However, the underlying issue of tumor heterogeneity cannot be neglected, as tumor cells are constantly evolving and remain in a plastic cell state. Therefore, future treatments will more than likely need to be combinatorial to target all subtypes and clones, as well as to be able to cross the BBB. As we look to the future of GBM treatments, areas that are particularly intriguing would be a better understanding of the GBM immune environment, as previous immunotherapies have not been successful, immune responses and cell types that may be unique to the brain could have an important impact on GBM treatment. Combination of chemotherapy with immune modulators, like TMZ with PD-1 blockade and CD47, and others could be an interesting avenue to study in the future [152]. Understanding the role of splicing factors and their mRNA products is also an exciting avenue of research, where mRNA therapies–like antisense oligonucleotides (ASOs)–are becoming more prevalent with our increased understanding of delivery methods. ASOs can be used to target certain splicing factors, as well as tumor-specific splicing events [153]. As the brain is the most alternatively spliced organ, it will be interesting to see the advances made to better understand and target aberrant splicing pathways in GBM [154]. Overall, more work is necessary to better understand the many facets of therapy-resistant GBM from both an intra- and inter-tumoral standpoint to create better second-line therapeutic options for patients diagnosed with recurrent GBM.

## Data Availability

No new data were created or analyzed in this study. Data sharing is not applicable to this article.
